# Pulmonary manifestations of childhood-onset primary Sjogren’s syndrome (SS) masquerading as reactive airways disease in a male patient and review of interstitial lung disease associated with SS

**DOI:** 10.1186/s12969-022-00761-z

**Published:** 2022-11-16

**Authors:** Adam Bartholomeo, Shean Aujla, Meryle Eklund, Cheryl Kerrigan, Ellen Riemer, Mileka Gilbert

**Affiliations:** 1 Department of Pediatrics, Medical University of South Carolina, 135 Rutledge Avenue, MSC 561, Charleston, SC 29425 USA; 2 Division of Pediatric Pulmonology, Allergy and Immunology, 125 Doughty Street, MSC 917, Charleston, SC 29425 USA; 3 Department of Radiology and Radiological Sciences, Medical University of South Carolina, 96 Jonathan Lucas St, MSC 323, Suite 210 CSB, Charleston, SC 29425 USA; 4 Department of Pathology and Laboratory Medicine, Medical University of South Carolina, 165 Ashley Avenue, Suite 337, Charleston, SC 29425 USA; 5 Division of Pediatric Rheumatology, Medical University of South Carolina, 125 Doughty Street, MSC 917, Charleston, SC 29425 USA

**Keywords:** Primary Sjogren’s syndrome, Pediatric patient, Interstitial lung disease, Mixed airway disease

## Abstract

**Background:**

Sjogren’s syndrome (SS) is a rare chronic autoimmune disease involving exocrine glands presenting with sicca syndrome, recurrent parotitis and other extraglandular stigmata. SS is well characterized in the adult population with classification criteria; however, primary SS presenting in childhood is poorly defined and rare in males. Recurrent parotitis is the most common presenting symptom in children with primary SS; however, clinical phenotype in children appears more variable than in adults. The lungs are a common extraglandular location for manifestations of primary SS. However, interstitial lung disease (ILD) is rare in children with primary SS. There are only four published reports of ILD associated with primary SS in female children. Here, we present a very rare case of primary SS in a pediatric male with pulmonary manifestations and review of the literature on ILD in childhood-onset primary SS.

**Case presentation:**

A 14-year-old White male with a history of chronic severe asthma, recurrent parotitis and idiopathic intracranial hypertension was referred to pediatric rheumatology for evaluation of a positive ANA. In early childhood, he was diagnosed with persistent asthma recalcitrant to therapy. At age 8, he developed recurrent episodes of bilateral parotitis despite multiple treatments with sialoendoscopy. At age 14, respiratory symptoms significantly worsened prompting reevaluation. Lab workup was notable for positive ANA and Sjogren’s Syndrome A and B antibodies. Pulmonary function tests showed only a mild obstructive process. Computed tomography of chest was significant for small airway disease, and lung biopsy was positive for mild interstitial lymphocytic inflammation presenting a conflicting picture for ILD. The constellation of findings led to the diagnosis of primary SS with associated pulmonary manifestations. He was treated with hydroxychloroquine, mycophenolate mofetil and oral corticosteroids with resolution of symptoms.

**Conclusions:**

Primary SS is a rare disease in the pediatric population that is poorly characterized. This case is the very rare presentation of childhood-onset primary SS with pulmonary manifestations in a male patient. ILD associated with primary SS is also very rare with only four pediatric patients reported in the literature. Collaborative effort is needed to develop pediatric specific diagnostic and treatment guidelines in this rare condition.

## Background

Sjogren syndrome (SS) is a rare chronic autoimmune disease involving exocrine glands and typically presents with sicca syndrome and recurrent parotitis, although extraglandular involvement may occur. Estimate of incidence of primary SS in adults is 3.9–5.3 cases per 100,000 persons with estimated prevalence of 43–282 per 100,000 persons in Europe [1]. Primary SS is more common in women than men with estimates of 9–13 to 1. The classification criteria for primary SS in adults were updated in 2016 by the American College Rheumatology (ACR) and the European League Against Rheumatism (EULAR) with clear data-driven consensus guidelines and weighted criteria [2]. However, given the purpose of classification criteria is standardized definitions for clinical research purposes, classification criteria should not be used for diagnosis in individual cases. There are no established classification or diagnostic criteria for childhood-onset SS.

SS presenting in childhood is poorly defined with unknown prevalence and prognosis [3]. Recurrent parotitis is the most common presenting symptom in children with SS (54–75% of cases), and children compared to adults present less frequently with dry eyes and mouth [4–7]. Clinical phenotype in children may be more variable than in adults, and thus classification criteria may not be adequate to diagnosis SS in the pediatric population [2, 6]. *Basiaga* et al showed that 77% of an international cohort of 300 pediatric patients with SS did not meet the ACR/EULAR classification criteria for SS, and only 36% of those who had all criteria items tested met criteria [8]. Thus, the ACR/EULAR classification criteria should not be used for the diagnosis of SS in childhood.

Extraglandular manifestations of SS occur in up to one third of pediatric and adult patients and include, but are not limited to, arthritis, myositis, rash, cytopenias, pulmonary involvement including interstitial lung disease (ILD), airway disease, and pulmonary hemorrhage, renal tubular acidosis, tubulointerstitial nephritis, and CNS involvement including optic neuritis, transverse myelitis, and vasculitis [6, 9–11]. ILD is common in adult patients with autoimmune connective tissue disease (CTD), however prevalence varies depending on type of CTD: rheumatoid arthritis (RA; 10–20%), systemic sclerosis (SSc; > 70%), SLE (1–15%), idiopathic inflammatory myopathies (20–78%), mixed connective tissue disease (MCTD; 53%) [10]. Nine to 20% of cases of SS have associated symptomatic pulmonary involvement and even more reported when systematically evaluated. Airway disease and ILD are the more common manifestations of pulmonary involvement in SS [9, 12–14].

There is a review of ILD associated with rheumatologic diseases in childhood, however the authors note only that primary SS is rare in children [15]. There are only four published reports, to our knowledge, of ILD associated with primary SS in children and only in females. Here, we report a very rare case of a pediatric male patient with pulmonary manifestations with possible interstitial lung involvement associated with primary SS [16].

## Case presentation

Patient presented to pediatric rheumatology clinic as a 14-year-old White male with history of poorly-controlled asthma, recurrent parotitis and idiopathic intracranial hypertension (IIH) referred for evaluation of a positive antinuclear antibody (ANA). Previously, he was diagnosed with asthma in preschool-age and had clinical response to therapy including inhaled corticosteroids. At age 8, he started to develop recurrent bilateral episodes of parotitis despite multiple treatments with sialoendoscopy with irrigation and steroid lavage by pediatric otolaryngology. At age 14, respiratory symptoms including wheeze, cough and shortness of breath particularly with exercise began to significantly worsen despite treatment with multiple asthma controllers, including inhaled corticosteroid and long-acting beta agonist, and allergy medications. This prompted further work up by pediatric pulmonology. Pulmonary function tests (PFTs) consistently showed an obstructive process (Table 1).

**Table 1:** ^a^Pulmonary function test results before and after treatment with immunosuppression

	Before Treatment	After 9 months of Treatment	After 3 years of Treatment
**FVC**	3.8 L (94% ref)	4.9 L (117% ref)	5.6 L (118% ref)
**FEV1**	2.8 L (80% ref)	3.6 L (99% ref)	4.1 L (100% ref)
**FEV1/FVC ratio**	73%	73%	73%
**FEF25–75**	2.2 L/s (56% ref)	2.8 L/s (71% ref)	3.3 L/s (72% ref)
**TLC**	5.8 L (120% ref)	6.5 L (129% ref)	7.6 L (142% ref)
**RV**	1.9 L (193% ref)	1.6 L (154% ref)	1.9 L (176% ref)
**RV/TLC ratio**	33%	25%	26%
**DLCO**	26.2 mL/mmHg/min (81% ref)	28.8 mL/mmHg/min (88% ref)	33.5 mL/mmHg/min (87% ref)
**Interpretation**			
**Spirometry**	Mild-moderate obstructive pattern	Mild obstructive pattern	Mild obstructive pattern
**Lung volumes**	TLC, RV and RV/TLC elevated	TLC and RV elevated	TLC and RV elevated
**DLCO**	Normal	Normal (improved from previous)	Normal (improved from previous)

^a^Abbreviations: *FVC* forced vital capacity, *FEV1* forced expiratory volume in 1 second, *FEF25–75* forced expiratory flow at 25–75% of FVC, *TLC* total lung capacity, *RV* residual volume, *DLCO* diffusing capacity for carbon monoxide, *L* liters, *s* second, *ref* reference, *mL/mmHg/min* milliliters/millimeters of mercury/minute

**Fig. 1:**
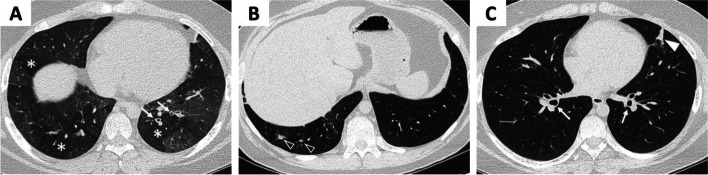
Pulmonary manifestations seen on computed tomography (CT) imaging of chest. Axial CT images obtained at 14 years of age demonstrate moderate mosaic attenuation of the lung parenchyma involving all lobes with scattered air trapping (asterisks, **A**) as well as bronchial wall thickening (white arrows, **A/C**). There are also clustered ground glass nodules (open arrowheads, **B**) and linear atelectasis (arrowhead, **C**), compatible with small airways disease

CT of chest was abnormal with evidence of significant small airway disease, bronchial wall thickening, ground glass nodules, and linear opacities in bilateral lower lobes consistent with atelectasis or scarring (Fig. 1). Bronchoscopy was notable for large amounts of purulent secretions with bronchoalveolar lavage cultures positive for *Haemophilus influenzae*. Ciliary biopsy was negative. He was treated with several courses of oral antibiotics targeting *H. influenzae* with some improvement but still suboptimal control of symptoms. Further testing included negative alpha-1 antitrypsin, negative angiotensin-converting enzyme level, and negative sweat chloride test with otherwise unremarkable immunoglobulin levels, mitogen titers (tetanus, diphtheria and pneumococcal) and total complement (CH50). However, ANA was positive with 1:160 titer prompting further evaluation by pediatric rheumatology. Concurrently, he continued to have persistent episodic parotitis, and a repeat CT sinus ordered by pediatric otolaryngology was unremarkable. Pediatric otolaryngology on report continued to treat symptomatically with repeat sialoendoscopy with steroid lavage as needed.

**Fig. 2:**
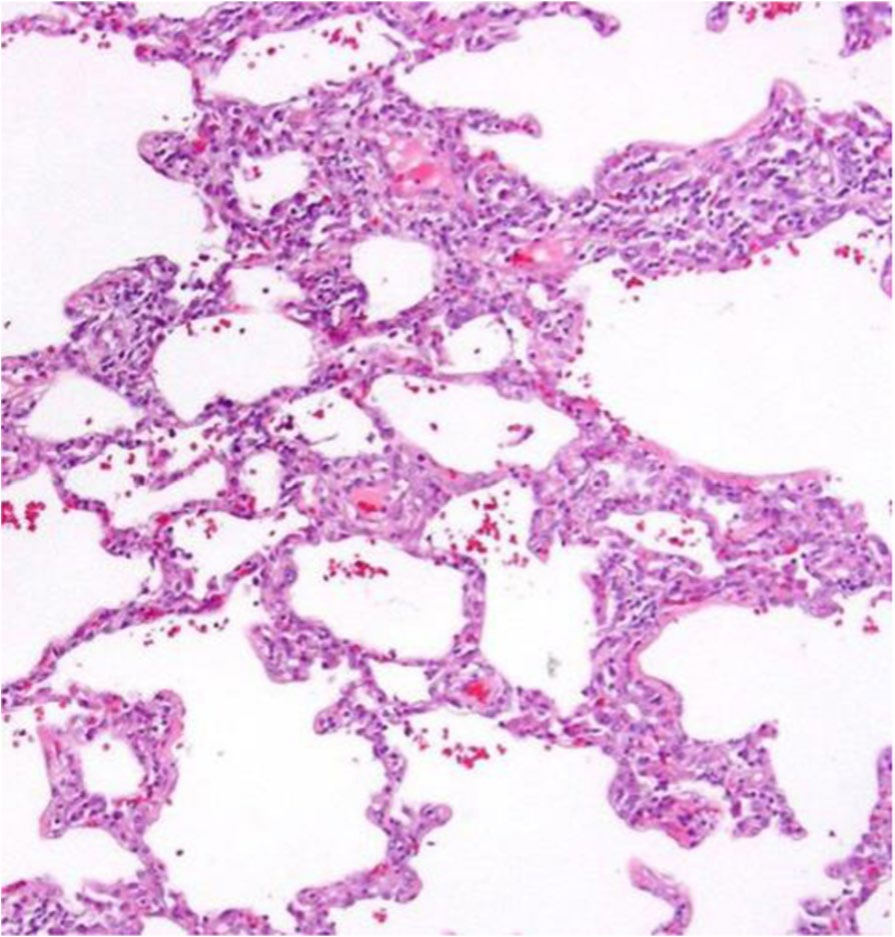
Histopathology showing the cellular type of nonspecific interstitial pneumonia (NSIP). Uniform alveolar septal thickening with small lymphocytes on hematoxylin and eosin staining of lung wedge biopsy

Review of systems was negative for fever, sicca symptoms, rash, easy bruising or bleeding, hemoptysis, bloody stools, abdominal pain, joint pain/swelling, weight loss, hair loss, and mouth sores. Additional pertinent positive symptoms included recurrent loose stools. There was no family history of autoimmune diseases. Physical exam was remarkable for coarse breath sounds and rhonchi, with unlabored breathing and prolonged expiratory phase, and mild joint hypermobility. There was no alopecia, oral ulcers, rash, skin tightening, and nailfold capillaroscopy was grossly normal. Initial rheumatology work up included repeat ANA 1:320 (speckled pattern) and positive Sjogren’s Syndrome A (SSA) and SSB antibodies. Rheumatoid factor, double-stranded DNA, ribonucleoprotein, and scleroderma-70 antibodies, complement 3 and 4 levels, and thyroid studies were normal or negative. Vasculitis work up, including antineutrophil cytoplasmic antibodies and urinalysis, was negative (Table 2). He underwent wedge resection biopsy of lingula with pathology positive for interstitial lymphocytic inflammation consistent with the cellular variant of nonspecific interstitial pneumonia (NSIP) pattern of chronic ILD (Fig. 2). At this point, the constellation of findings, including recurrent parotitis, recalcitrant poorly-controlled asthma with significant small airway disease on HRCT, NSIP pattern on lung wedge biopsy, and IIH in the context of a positive ANA and SSA/SSB, were consistent with a clinical diagnosis of primary SS associated with mixed airway and pulmonary parenchymal disease.

**Table 2:** Laboratory evaluation before and after treatment with immunosuppression

Laboratory Test	Before Treatment	6 Months After Treatment	Reference range
**White blood count**	7.21	7.78	4.8–10.8 × 10^3^/mm^3^
**Hemoglobin**	14.0	13.7	14.0–18.0 g/dL
**Platelet count**	317	369	140–440 × 10^3^/mm^3^
**Erythrocyte sedimentation rate**	9	14	0–15 mm/hr
**Aspartate aminotransferase**	24	16	14–35 U/L
**Alanine aminotransferase**	17	14	9–24 U/L
**Serum albumin**	3.9	4.0	3.8–5.0 g/dL
**Serum creatinine**	0.7	0.8	0.6–1.2 mg/dL
**Complement 3**	179	109	83–152 mg/dL
**Complement 4**	25	19	13–37 mg/dL
**Serology**	**ANA 1:320 speckled**	**ANA 1:160 speckled**	Negative
	dsDNA Ab negative	dsDNA Ab negative	Negative < 0.9
	**+SSA Ab > 8**	**+SSA Ab > 8**	Negative < 0.9
	**+SSB Ab 1.8**	SSB Ab negative	Negative < 0.9
	RNP Ab negative	RNP Ab negative	Negative < 0.9
	Smith Ab negative	Smith Ab negative	Negative < 0.9
	ANCA negative		Negative
**Urinalysis**	Negative hematuria, proteinuria	Negative hematuria, proteinuria	Negative

He was started on treatment with high-dose oral prednisone taper over 3 months, hydroxychloroquine (HCQ), and mycophenolate mofetil (MMF) with gradual improvement in all symptoms overtime with only residual occasional cough. He had no persistent wheezing or shortness of breath, and exercise tolerance improved. He had no further episode of parotitis. He became active in Taekwondo. He was able to taper off acetazolamide for IIH and wean down on asthma medications without worsening chest symptoms. Flows on spirometry improved, although he still had evidence of air trapping with elevated total lung capacity (TLC) and residual volume (RV) on PFTs (Table 1). Repeat CT of chest showed marked improvement in lung disease. Subsequent tonsillectomy for sleep apnea and enlarged tonsils revealed benign lymphoid follicular hyperplasia without concern for lymphoma. Given his overall positive response to therapy, a clinical diagnosis of primary SS was felt sufficient and, as such, further invasive testing with salivary gland biopsy or imaging were not pursued.

## Discussion

The pediatric patient presented here demonstrates multiple manifestations of SS, including recurrent parotitis, IIH, and lung disease with positive ANA, SSA and SSB antibodies, without sicca symptoms. His ANA titer 1:320 (titers > 1:160 occur in only 5% of healthy individuals [17]) and positive SSA and SSB antibodies, along with the multiple chronic clinical features of SS, were highly suggestive of SS diagnosis despite not meeting the ACR/EULAR classification criteria. Our patient presented with recurrent parotitis, like the majority of pediatric patients with SS, although 6 years prior to his diagnosis of SS. While the differential diagnosis of recurrent parotitis is broad including mechanical (stones, stricture), viral or bacterial infection, malignancy, autoimmune (SS, sarcoidosis), or idiopathic, more children than adults with primary SS present with recurrent parotitis. Thus, primary SS must be considered in a child or adolescent that presents with recurrent parotitis.

This patient’s early pulmonary symptoms included airway hyperresponsiveness as reported in 42–60% of adult patients with primary SS [9]; however, worsening pulmonary symptoms in adolescence after the development of recurrent parotitis led to re-evaluation of his asthma diagnosis. While PFTs and CT were not completely consistent with ILD (Fig. 1), histopathology suggested the NSIP type of ILD (Fig. 2). Additionally, respiratory symptoms responded very well to immunosuppression, suggesting an autoimmune etiology. Thus, his evaluation presents a conflicting picture for ILD.

There are only four previously published reports, to our knowledge, of ILD associated with primary SS in children. A summarizing description of each of these patients as reported in the literature is outlined in Table 3. Our patient is noted to have some significant key features that are unique compared to these other four patients with ILD associated with primary SS. First, ILD associated with primary SS presenting in a male pediatric patient is notable as this disease has a female predominance. In fact, this is the only male pediatric patient reported with possible ILD associated with primary SS to our knowledge. Second, our patient developed significant respiratory symptoms at an early age, diagnosed as asthma based on symptoms and obstructive process seen on PFTs (at one point having a significant bronchodilator response) years before the onset of recurrent parotitis associated with SS. However, worsening and refractory respiratory symptoms upon reaching adolescence prompted the initial rheumatologic evaluation. This is unlike the other patients who appear to have developed pulmonary involvement concurrent or subsequent to sicca symptoms or parotitis. Two cases appear to have initially presented with recurrent parotitis and sicca symptoms prior to documented pulmonary involvement [7, 19]. Sicca symptoms for our patient were never central or persistent. The youngest patient in *Vermylen* et al appears to have developed recurrent pneumonia and noticeable parotitis concurrently [18], but the other two patients did not have documented respiratory symptoms until adolescence [7, 19]. In the final patient in *Tomiita* et al, the timeline between presenting symptoms and development of lung findings is unclear given data is presented as an aggregate [20]. It is possible that our patient has two pulmonary diagnoses: asthma diagnosed at preschool age and then SS-related disease in adolescence after presenting with recurrent parotitis more consistent with cases reported in the literature. Third, our patient also had concomitant neurologic involvement with IIH associated with primary SS which was not present in the reported cases.

**Table 3:** ^a^Description of patients with childhood-onset primary Sjogren’s Syndrome associated with interstitial lung disease

Reference	Presenting Symptoms	Additional Positive Clinical features	Positive Lab Findings	Imaging	Biopsy	Treatment	Outcomes
**Our Patient**	14 yo White male with recurrent parotitis and wheezing, dyspnea, and cough recalcitrant to asthma therapy	IIH Exercise intolerance Joint hypermobility	ANA 1:320 (speckled pattern) SSA/SSB Low Forced Vital Capacity on PFTs	CT with significant small airway disease, bronchial wall thickening, ground glass nodules	Interstitial lymphocytic inflammation on lung wedge biopsy (NSIP)	Systemic oral steroids and MMF therapy	Significant clinical, radiographic and PFTs improvement and maintained on MMF
**Vermylen C 1985 Eur J Pediatr** [18]	5 yo Black female with recurrent respiratory infections including rhinopharyngitis and pneumonia, and failure to thrive	Parotitis, cervical lymphadenopathy, cheilitis, diffuse rhonchi and rales, mild hepatomegaly	Anemia, Hypergammaglobulinemia, elevated sedimentation rate/C-reactive protein	CXR with diffuse interstitial infiltrations, echography of parotids showing enlargement with sialectasia	Parotid biopsy with intra- and periductal lymphocytic infiltration	Systemic steroids	Clinical improvement within 2 weeks with complete resolution of parotid swelling and normalization lab findings
**Anaya JM 1995 J Rheumatol** [19]	24 yo Hispanic woman with 14 years fever, migratory arthralgia, cervical adenopathy and recurrent parotitis	Xerostomia, dental carries, left eye synechia and positive Schirmer test	Hypergammaglobulinemia, RF, ANA, SSA/SSB, and anemia DLCO showed 60% of predicted	CXR was clear	Minor salvatory biopsy with focal lymphocytic sialadenitis	Supportive and 1 year systemic steroids	Persistent parotid swelling and xerostomia without development of systemic findings
**Houghton KM 2005 J Rheumatol** [7]	14 yo dizygotic-twin Vietnamese-Canadian girl with cough, dry mouth, SOB with 4 episodes parotitis starting at age 3. Frequent dental carries and dental abscesses.	Cervical lymphadenopathy; mild clubbing, diffuse crackles and decrease aeration at base on lung exam; right keratitis; Dizygotic twin sister with primary SS	RF, ANA, SSA/SSB, anemia, leukopenia and hyperglammaglobulinemia Elevated FEV1/FVC ratio and decreased DLCO by 80%	CXR with multiple areas of consolidation; CT lungs showed multiple parenchymal densities with air bronchograms	Lung biopsy showing bronchial with predominant lymphocytic infiltrate	High-dose pulse IV steroids with course of oral systemic steroids and HCQ	Clinical and radiographic improvement in symptoms
**Tomiita M 1997 Acta Paediatr Jpn** [20]	61 cases reported with 52 girls and 6 boys with initial onset of symptoms ranging from ages 3 to 14. 70% noted to have primary SS w/ common symptoms including fever, sicca symptoms, recurrent parotitis, lymphadenopathy, and arthralgia	Interstitial pneumonia noted in 1 patient	ANA, hypergamma-globuminemia, RF, SSA/SSB were observed	None reported	All showed lymphocytic infiltration in a minor salivary gland biopsy	None reported	None reported

^a^Abbreviations: *yo* year old, *ANA* antinuclear antibody, *SS* Sjogren’s Syndrome, *CT* high-resolution computed tomography, *PFTs* pulmonary function tests, *NSIP* nonspecific interstitial pneumonia, *MMF* mycophenolate mofetil, *CXR* chest radiograph, *RF* rheumatoid factor, *DLCO* diffusing capacity of carbon monoxide, *FEV1/FVC* forced expiratory volume in 1 second/forced vital capacity, *HCQ* hydroxychloroquine

Lastly, our patient with primary SS demonstrates heterogenous lung features. He had a clear obstructive process present on PFTs (decreased FEV1/FVC ratio and decreased FEF25–75%) suggesting diagnosis of asthma/airway disease initially. However, his progression of symptoms and eventual lack of response to asthma therapy was clearly out of proportion to these findings, suggesting need for further evaluation. In fact, despite having obstructive pattern on PFTs and small airways disease on chest CT, our patient had NSIP on lung wedge biopsy consistent with co-existing subclinical parenchymal disease. Again, in *Vermylen* et al the patient’s lung features were primarily infectious with recurrent pneumonia [18], while the adolescents in both *Anaya* et al and *Houghton* et al had a primarily restrictive component with decreased diffusing capacity for carbon monoxide (DLCO) [7, 19]. *Houghton* et al was the only other patient to show lymphocytic infiltrate on lung biopsy, but this correlated clinically with restrictive pattern [7], while our patient had evidence of obstructive process. As evident in adult data, abnormal PFTs in patients with SS more often reveal restrictive (parenchymal involvement) rather than obstructive pattern (airway involvement), although it is important to note that both patterns do co-exist. Therefore, in early disease progression or subclinical pulmonary disease, PFTs may not classically show a restrictive pattern. While reduced DLCO is the most common abnormality, it may be an insensitive test for subclinical pulmonary disease in this case. Interestingly, our patient did not have abnormal DLCO or restriction (despite NSIP on lung wedge biopsy) but did have evidence of air trapping on lung volumes (elevated RV and TLC), consistent with obstructive airway disease. This is not surprising in light of the bronchial involvement present on chest CT. Increased prevalence of chronic obstructive pulmonary disease has been described in primary SS patients [21].

Given primary SS in children is poorly characterized at this point in time, this unique case of primary SS with secondary lung features in context of the other reported cases in the literature emphasizes the degree of variability in the clinical presentation of primary SS in children. It also demonstrates the need to better distinguish childhood-onset SS from adult-onset SS to allow for earlier and more accurate diagnosis in childhood. It is possible that lung features may be the initial presentation of CTD, although this is not typical of CTD with lung disease such as ILD. In the case of inflammatory lung disease without a known diagnosis of CTD, evaluation for clinical features of CTD, including sicca symptoms, Raynaud’s, sclerodermatous skin changes, rash, myositis, and arthritis, in addition to evaluation for infection is necessary. Bronchoscopy for evaluation of bronchoalveolar lavage fluid may be helpful depending on clinical scenario. Chronic respiratory symptoms, such as dyspnea, cough, crackles, clubbing, in any patient with known CTD including primary SS should prompt evaluation of lung involvement with PFTs (spirometry, complete lung volumes and DLCO) and, if abnormal, with CT. The added concern is that some drugs used for CTD can cause lung pathology, such as ILD, including methotrexate, TNF blocking agents, gold, penicillamine, leflunomide, and sulfonamide [12]. Lung biopsy is recommended if the underlying diagnosis is uncertain, particularly if there is concern for malignancy. There is a potential role for lung biopsy to determine histological pattern and type of lung disease or ILD and help predict prognosis [12]. However, CT pattern correlates with histological pattern particularly in the NSIP type of ILD [9]. The potential benefits of lung biopsy must be weighed against the potential risks of this surgical procedure.

Chest radiograph can show bilateral lung infiltrates with linear and reticular opacities in 10–30% of SS patients with lung involvement. CT is the most sensitive imaging study for detecting lung disease in 31–90% of SS patients, showing ground glass opacities, non-septal linear opacities, interlobular septal thickening and cysts, reticulation, and fibrosis [9].

There is no robust pediatric specific data for treatment of childhood-onset CTD with ILD, and current treatment options are based on treatment for CTD-associated ILD in adults; however, there are no specific guidelines for treatment of CTD-associated ILD either. Treatment of chronic presentations of CTD-ILD has been extrapolated from adult randomized-controlled trials (RCTs) of drug therapies for scleroderma-associated ILD and Idiopathic Pulmonary Fibrosis (IPF) and adult case series of patients with ILD associated with SSc, polymyositis (PM), dermatomyositis (DM), and MCTD [12]. Corticosteroids and an immunosuppressive agent are the mainstay of therapy for CTD-ILD. Patients with SSc-related ILD treated with oral cyclophosphamide (CYC) in the double-blind, randomized, placebo-controlled Scleroderma Lung Study (SLS)-I demonstrated better lung function after 12 and 24 months [22]. SLS-II was a double-blind, parallel group, RCT that showed MMF treatment over 2 years compared to oral CYC over 12 months led to similar efficacy after 2 years in scleroderma-related ILD, although MMF had better tolerability and toxicity profile [23]. However, a case-controlled study of small numbers of patients with SSc-ILD treated with MMF or oral or intravenous CYC showed no difference in lung function and possible worsening of radiographic findings with MMF [24]. A few case series and retrospective review support the use of MMF in ILD associated with SS-ILD, PM or DM-ILD, and RA-ILD with reported sustained improvement in FVC [25–28]. *Mathai* et al also reviewed the use of azathioprine, calcineurin inhibitors, rituximab, and emerging therapies in CTD-ILD. Methotrexate and TNF blocking agents are used less frequently in CTD-ILD due to concern for pulmonary toxicity as an adverse effect [12].

Prednisone taper, HCQ and MMF were chosen for treatment of SS-ILD in our patient presented here due to results of the SLS-II. FVC and CT results in our patient in combination with significant clinical improvement in symptoms demonstrate that MMF was an effective immunosuppressant agent in the treatment of pSS with lung involvement [23]. Moreover, the robust positive response to prednisone taper and MMF suggests underlying autoimmune etiology with possible ILD. Further research in pediatric populations is necessary in order to develop specific guidelines for treatment of CTD with ILD in children.

## Conclusion

Primary SS is a very rare disease in the pediatric population but must be considered in a child or adolescent presenting with recurrent parotitis. This case is the very rare presentation of childhood-onset primary SS with pulmonary manifestations (with possible ILD) in a male patient. Respiratory symptoms, FVC, and chest CT improved after treatment with corticosteroid taper and MMF therapy similar to patients with SSc-related ILD. ILD associated with primary SS is also very rare with only four pediatric patients previously reported in the literature. Collaborative effort is needed to develop pediatric specific diagnostic and treatment guidelines in this rare condition.

## Data Availability

Not applicable.

## Abbreviations

ACR: American College of Rheumatology
ANA: Antinuclear antibody
CTD: Connective tissue disease
CXR: Chest radiograph
CYC: Cyclophosphamide
DLCO: Diffusing capacity for carbon monoxide
DM: Dermatomyositis
EULAR: European League Against Rheumatism
FEF25–75: Forced expiratory flow at 25–75%
FEV1: Forced expiratory volume in 1 second
FVC: Forced vital capacity
HCQ: Hydroxychloroquine
CT: Computed tomography
IIH: Idiopathic intracranial hypertension
ILD: Interstitial lung disease
IPF: Idiopathic Pulmonary Fibrosis
MCTD: Mixed connective tissue disease
MMF: Mycophenolate mofetil
NSIP: Nonspecific interstitial pneumonia
PFT: Pulmonary function test
PM: Polymyositis
RA: Rheumatoid arthritis
RCT: Randomized controlled trial
RF: Rheumatoid factor
RV: Residual volume
SS: Sjogren’s syndrome
SSc: Systemic sclerosis
TLC: Total lung capacity
